# Designed Antimicrobial Peptides Against Trauma-Related Cutaneous Invasive Fungal Wound Infections

**DOI:** 10.3390/jof6030184

**Published:** 2020-09-22

**Authors:** Kathryn W. Woodburn, Jesse M. Jaynes, L. Edward Clemens

**Affiliations:** 1 Riptide Bioscience, Vallejo, CA 94592, USA; eclemens@riptidebio.com; 2 Integrative Biosciences, Tuskegee University, Tuskegee, AL 36088, USA; jjaynes@tuskegee.edu

**Keywords:** antimicrobial peptides, host defense peptides, cutaneous invasive fungal wound infections, topical treatment, wound infections

## Abstract

Cutaneous invasive fungal wound infections after life-threatening dismounted complex blast injury (DCBI) and natural disasters complicate clinical care. These wounds often require aggressive repeated surgical debridement, can result in amputations and hemipelvectomies and have a 38% mortality rate. Given the substantial morbidity associated with cutaneous fungal wound infections, patients at risk need immediate empiric treatment mandating the use of rapidly acting broad-spectrum antimicrobials, acting on both fungi and bacteria, that are also effective against biofilm and can be administered topically. Designed antimicrobial peptides (dAMPs) are engineered analogues of innate antimicrobial peptides which provide the first line of defense against invading pathogens. The antifungal and antibacterial effect and mammalian cytotoxicity of seven innovative dAMPs, created by iterative structural analog revisions and physicochemical and functional testing were investigated. The dAMPs possess broad-spectrum antifungal activity, in addition to being effective against Gram-negative and Gram-positive bacteria, which is crucial as many wounds are polymicrobial and require immediate empiric treatment. Three of the most potent dAMPs—RP504, RP556 and RP557—possess limited mammalian cytotoxicity following 8 h incubation. If these encouraging broad-spectrum antimicrobial and rapid acting results are translated clinically, these novel dAMPs may become a first line empiric topical treatment for traumatic wound injuries.

## 1. Introduction

Combat-related cutaneous invasive fungal wound infections cause substantial morbidity with mortality ranging from 11% to 38% [1,2,3]. Cutaneous invasive fungal infections are mostly attributed to dismounted complex blast injury (DCBI), where those on foot patrol experience environmental contamination of their blast wounds [4]. Local geography has necessitated dismounted patrols in many combat zones, placing military personnel at substantial risk for trauma-related infections [2,5]. Severe DCBI is characterized by traumatic injuries to the lower extremities, causing amputation of at least one leg, and/or upper extremity, in addition to pelvic injuries, genitourinary and abdominal trauma [6]. DCBIs are highly susceptible to both bacterial and fungal infections [7]. The blast exposure provides a traumatic penetrating pathway for deep implantation of pathogenic environmental, particularly soil, organisms into soft tissue [8]. The culprit molds in conflict regions, such as southern Afghanistan, are the Mucorales, *Aspergillus* and *Fusarium* species [6], with associated co-infection with bacteria such as *Enterococcus* spp., *Acinetobacter baumannii* and *P. aeruginosa* [9]. Moreover 9% of wounds have tested positive for *Candida albicans* [10]. Trauma-related cutaneous fungal infections also occur after natural disasters; 38% of the cases diagnosed from the 2011 tornado in Joplin, Missouri, resulted in death [11].

Fungal infections adversely impact wound healing and patient recovery, requiring frequent repeated amputations and substantial early complications [8]. The high mortality rates underscore the need for appropriate diagnosis and initiation of aggressive surgical intervention with concomitant empiric antimicrobial treatment. Repeated aggressive surgical debridement is the main treatment for these patients [12], with empiric systemic antifungal therapy and topical application of Dakin’s (hypochlorite) solution [13]. Empiric anti-infective treatment is needed as the median time from injury to fungal diagnosis is 10 days [9]. Presently used antifungal agents are often ineffective in treating cutaneous invasive fungal infections so further aggressive debridement into clinically viable tissue is undertaken which can result in severely disabling procedures, such as extremity amputation, hip disarticulation and hemipelvectomy [14].

Given the limited treatment options for trauma-related fungal infections and the severity of the injuries and dismal outcomes, there is an understated unmet medical need for adjunctive topical antifungal wound agents. More so as traumatic wounds, especially burns, have limited bioavailability following systemic antibiotic exposure due to marked vascular damage. Innovative topical antifungals that are effective against a broad-spectrum of fungal infections, including Mucorales, *Aspergillus* spp. and *Fusarium* spp. and are also active against wound co-inhabited bacteria are needed for surgical and post-surgical treatment of DCBIs so infections can be prevented.

Antimicrobial peptides (AMPs), also known as host defense peptides, are evolutionarily highly conserved components of the innate immune system and provide the first line of defense against invading pathogens [15]. Designed antimicrobial peptides (dAMPs) are laboratory synthesized peptides which have been rationally designed from naturally occurring AMPs [16]. AMPs have direct and immediate anti-pathogenic activities, killing fungi and both Gram-negative and Gram-positive bacteria [17,18], in addition to modulating immune responses [16]. AMPs generally act by interacting electrostatically and perturbing the barrier function of the pathogen’s membrane [17], complexing with ergosterol, a membrane lipid almost exclusively found in fungi, and a sterol present in conidial walls, thereby leading to lytic membrane disruption [19], conferring selective targeting compared to mammalian cells which contain more zwitterionic phospholipids framed with cholesterol and cholesterol esters [20]. Moreover, this remarkable targeting and direct contact disruption of the pathogen’s membrane makes resistance less likely to develop [16,17,18]. Furthermore, dAMPs, exhibit potent activity against both recalcitrant fungal and bacterial biofilm [17,18].

We previously have identified the 17 amino acid disulfide peptide dAMP, RP557 as a potent fast acting broad-spectrum anti-infective with limited potential for pathogen resistance [17,18]. Here the utility of RP557 will be evaluated with the earlier generation 17 amino acid linear peptide D4E1, and six comparator innovative dAMPs in representative antifungal and Gram-positive and Gram-negative bacteria in vitro assays. Mammalian cytotoxicity will be assessed using L929 fibroblast cells. As immediate acting empirical treatment is crucial to offset morbidity and mortality, *P. aeruginosa* and *S. aureus* time-kill evaluation of the most promising peptides, in comparison to RP557 [17,18], were performed to demonstrate the potential utility of dAMPs for trauma-related cutaneous invasive fungal wound infections.

## 2. Materials and Methods

### 2.1. Designed Antimicrobial Peptides

**Figure 1:**
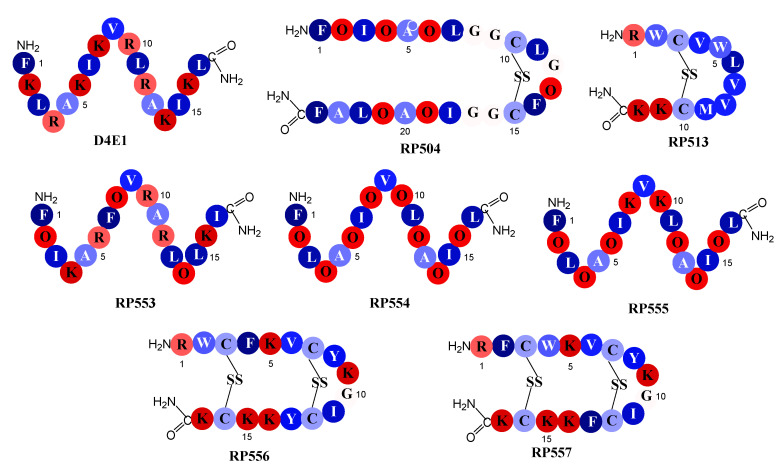
Schematic relative hydrophobicity representation of the designed antimicrobial peptides. Single letter codes of the amino acids are depicted with dark blue representing the most hydrophobic amino acid, phenylalanine (F), through to the hydrophilic amino acids in red, lysine (K). Relative hydrophobicity represented by the energy, kcal/mole, necessary to move an amino acid from an aqueous phase to a lipid bilayer [22]: F, phenylalanine, −3.85; L, leucine, −3.36; I, isoleucine, −3.16; Y, tyrosine, −2.66; M, methionine, −2.34; V, valine, −2.34; W, tryptophan, −1.96; A, alanine, −1.56; C, cysteine, −1.06; G, glycine, −0.14; R, arginine, 2.22; O, ornithine, 3.56; and lysine, K, 3.85.

The seven novel dAMPs and the reference dAMP, D4E1, whose amino acid sequences are depicted in Figure 1, were synthesized via solid phase synthesis (AmbioPharm, North Augusta, SC, USA). Peptide purity was >96% as assayed by high performance liquid chromatography and mass spectroscopy. The dAMPs were tailored to improve upon both natural and synthetic AMP libraries. The structures were generated using computational chemistry PyMol software (Schrodinger, Cambridge, MA, USA) and analyzed using the antimicrobial peptide predictor tools: Antimicrobial Peptide Database with the APD3 algorithm [19].

The reference peptide D4E1 is an early generation novel dAMP whose sequence is based upon the naturally occurring class of host defense peptides, the β-defensins. [21] Members of this defensin family are 2–6 kDa and have three pairs of intramolecular disulfide bonds whereas D4E1 is a 2 kDa linear β-sheet that has retained the parent cationic charge, amphipathic characteristics and microbicidal activity. The amino acid sequences in peptides RP553, RP554 and RP555 follow the same pattern of alternating cationic amino acids and hydrophobic amino acids as described in D4E1. To alter the hydrophobicity and conformation of the peptides the non-natural amino acid ornithine was substituted for lysine or arginine in some positions. The inclusion of ornithine increased antibacterial activity and enhanced proteolytic stability; however, the generated peptides produced a narrow Therapeutic Index. The second and third iterations evaluated refinements in conformational and amphipathic characteristics, optimized hydrophobic moments, and modified the centers of cationic charge associated with Tachyplesin 1 by exchanging arginine for lysine. The unique sequences of RP504, RP556 and RP557 provide a relationship between net charge, amphipathicity and hydrophobicity that has resulted in decreased proteolytic degradation, broad-spectrum antimicrobial and biofilm inhibition activity with reduced mammalian cytotoxicity and an increased Therapeutic Index.

### 2.2. Pathogens

Fungal and bacterial strains were obtained from American Type Culture Collection (ATCC, Manassas, VA, USA) or the clinical isolate collection at Trideum Biosciences (Frederick, MD, USA) and are presented in Table 1 and Table 2, respectively. Bioluminescence strains of *P. aeruginosa* 19660, transfected with the Xen5 luciferase gene, and *S. aureus* ATCC R 49525TM (Wright), transfected with the Xen36 luciferase gene, were obtained from Perkin Elmer (Waltham, MA, USA).

### 2.3. Minimum Inhibitory Concentration (MIC) Assays

Assays were performed by Trideum Biosciences (Frederick, MD, USA). Isolates were thawed from −80 °C storage and sub-cultured on trypticase soy agar plates at 37 °C prior to experimentation. A single colony was aseptically picked from the agar plate and released in 30 mL trypticase soy broth and incubated overnight at 37 °C incubator. Following which the bacterial culture was diluted to approximately 10^6^ colony forming units (CFU)/mL using 2X cation adjusted Mueller–Hinton broth and added to the dAMP solutions (0, 2, 4, 8, 16, 32, 64, and 128 μg/mL) which had been formulated in water at 256 μg/mL and then serially diluted in media on 96-well pates. For fungal evaluation, potato dextrose broth and potato dextrose agar plates were used. The treated cultures were then incubated for 24 h at 37 °C and pathogen growth was measured by absorbance at 600 nm. Four replicas were included for each dAMP concentration. Each MIC was assigned to the lowest test agent concentration resulting in at least three out of four wells showing no growth based on an optical density (OD) reading of <0.1.

### 2.4. Bioluminescent Time-kill Assays

Real-time killing of bacterial cells, *P. aeruginosa* or *S. aureus*, and mammalian cytotoxicity to assess dAMP off-target toxicity was evaluated using bioluminescent cells. The L929 fibroblast cells (ATCC, Manassas, VA, USA) were made bioluminescent by transfection with a luciferase gene [18]. Cells (1 × 10^4^ cells, 100 μL) were plated in 96-well black-walled plates and the candidate dAMPs were 2-fold serially diluted from specified concentrations in growth medium supplemented with 150 μg/mL d-luciferin. Each concentration was performed in triplicate, with the final volume being 200 μL. Imaging was performed at select times after the addition of the dAMP, and compared to concurrently run vehicle-control, using an IVIS Lumina imaging system (Caliper Life Sciences, Inc., Hopkinton, MA, USA). For imaging, the 96-well plate was positioned on the stage (12.5-cm field of view), with an open emission filter, binning of 4, and f-stop 1 and a 1-min exposure time. Monitoring bioluminescence allows assessment of the kinetics of cellular viability as there is a tight correlation between the bioluminescent signal and cell viability [23]. Data analysis was performed using the Living Image software program (version 4.3, Caliper Life Sciences, Inc., Hopkinton, MA, USA).

### 2.5. Statistical Analysis

Quantitative data were expressed as mean ± standard error of the mean. Statistical analysis was performed using GraphPad Prism 7 (GraphPad Software, San Diego, CA, USA). Comparisons were performed using a 1-way analysis of variance (ANOVA) followed by a post hoc Dunnett’s test. A *p* value < 0.05 was considered statistically significant.

## 3. Results

### 3.1. dAMP Antifungal Activity

Seven innovative dAMP sequences, including the precursor D4E1, were screened for in vitro antifungal activity, doses ranging from 2 to 128 μg/mL, against several fungal strains. All evaluated dAMPs, except for the 12 amino acid RP513, exhibited better activity than D4E1 (Table 1). The 24-amino acid disulfide dAMP, RP504, though effective against Candida strains did not exhibit appropriate inhibitory activity against the main cutaneous invasive fungal infection culprit species, Mucorales and Aspergillus though was active against Fusarium and Candida albicans. A substitution of the un-natural amino acid ornithine at positions 8 and 10 for lysine to form RP554, from RP555, resulted in a marked improvement in activity (OVO sequence at positions 8, 9 and 10 compared to KVK in RP554). The dAMPs, RP554, RP556 and RP557 all possessed similar potent broad-spectrum activity in eradicating the evaluated fungal strains.

### 3.2. dAMP Antibacterial Activity

The dAMPs, with exception of the 12 amino acid dAMP RP513, possess broad-spectrum antimicrobial activity against several bacterial strains including the most reported coinfecting bacterial pathogens in trauma wounds *P. aeruginosa*, *A. baumannii* and *S. aureus*, and ESKAPE pathogens (*Enterococcus faecium, Staphylococcus aureus, Klebsiella pneumoniae, Acinetobacter baumannii, Pseudomonas aeruginosa,* and *Enterobacter spp.*) (Table 2). This broad-spectrum empiric microbial activity is advantageous when treating recalcitrant mixed-species infections [9]. Unlike the activity profile for fungi, RP554 was not markedly better than RP555. The disulfide dAMPs (RP556 and RP557) both exhibited better activity compared to the other evaluated counterparts.

### 3.3. Limited Mammalian Cell Toxicity

**Figure 2:**
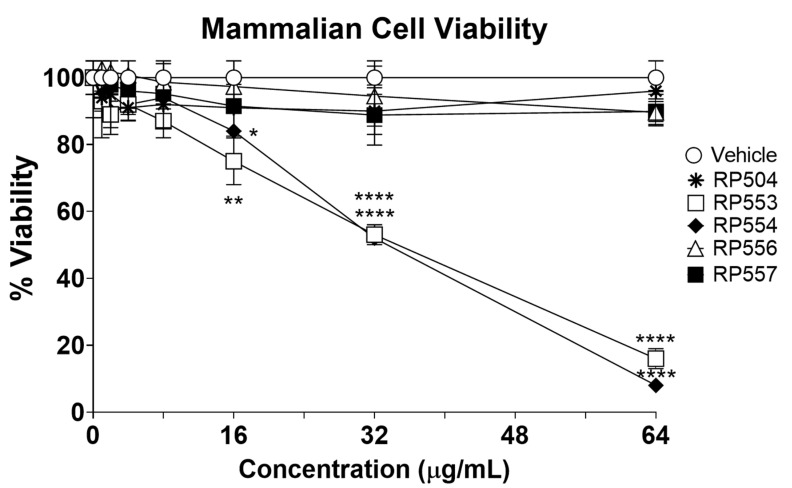
RP504, RP556 and RP557 induce limited mammalian cell toxicity. Concentration dependent viability analysis of murine L929 fibroblast cells following 8 h incubation. Cells were plated at 1×10^4^ cells/well, allowed to adhere overnight and the specific dAMP added, and cytotoxicity evaluated through 8 h. Cellular toxicity was assayed using a bioluminescent strain of fibroblasts and viability assayed using an IVIS Lumina imaging system (Perkin Elmer). Data shown represent the mean of triplicate replicates; statistically significant (* *p* < 0.05, ** *p* < 0.01, **** *p* < 0.0001), using one-way ANOVA followed by Dunnett’s analysis. For some points, the error bars are shorter than the height of the symbols.

The dAMPs under investigation, using an innovative real-time in vitro bioluminescent time-kill assay, demonstrated marked differences in killing mammalian (potential host) cells. To confirm selective activity, mammalian cell cytotoxicity was assessed using fibroblasts up to 64 μg/mL following 8 h of incubation. The concentration-dependent cytotoxic effects of each of the most promising anti-infective dAMPs evaluated here are displayed Figure 2. Limited toxicity was observed for RP504, RP556 and RP557 following 8 h of exposure with approximately 20% viability obtained with RP553 and RP554 at 64 μg/mL. A combination of the charge and substantially reduced hydrophobicity provided by the ornithine, in concert with the linear structure is thought responsible for the mammalian cell cytotoxicity induced by RP553 and RP554.

**Figure 3:**
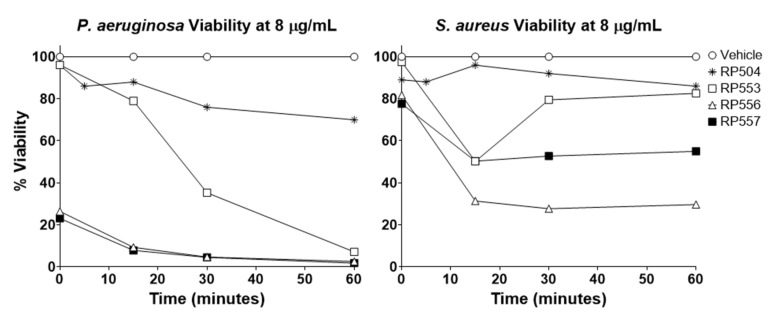
RP553, RP556, RP557 rapidly destroy P. aeruginosa and S. aureus. The bioluminescence of viable *P. aeruginosa* 19,660 and *S. aureus* 49,525 cells was quantitated in real time with an IVIS Lumina bioimaging system. Data represent the mean ± SE of triplicate replicates from two independent experiments. For some points, the error bars are shorter than the height of the symbols.

As many wounds are polymicrobial in nature, and empiric rapid treatment is preferred, antibacterial time-kills assays were performed using bioluminescent *P. aeruginosa* and *S. aureus* (Figure 3). RP556 and RP557 caused immediate eradication of *P. aeruginosa* following 8 μg/mL, with RP553 destroying nearly all the pathogen at 60 min with RP504 destroying 25%. For *S. aureus*, following 60 min incubation at 8 μg/mL, RP557, RP556, RP553, and RP504 induced 70, 45, 14, and 13% killing, respectively.

## 4. Discussion

Cutaneous invasive fungal infections following traumatic injury can result in 38% mortality [1,2,3]. Treatment of severe infection involves radical surgical debridement and, in some cases, hemipelvectomy or hip disarticulation are required. These infection-induced surgical procedures reduce rehabilitative potential by limiting the use of lower extremity prosthetics to restore ambulation. Due to the rapid pace of disease progression and limited evidence supporting the efficacy of systemic antifungal therapies for this disease, surgical debridement remains the primary therapeutic modality. The increasing emergence of antibiotic resistance highlights the need for innovative alternatives that provide rapid and complete microbicidal activity with minimal safety-related effects, while exhibiting limited susceptibility to microbial resistance. Therefore, development of new medical countermeasures, including topical approaches, are urgently needed to improve the outcome of these infections.

The designed antimicrobial peptides evaluated here are synthetic peptides derived from both natural and synthetic AMP libraries [16,17,18,24]. Iterative evaluation was utilized to obtain optimal anti-pathogenic function, rationally designed using conformational topology for potency to the pathogen target (both fungal and bacterial) while limiting toxicity to mammalian cells (Table 1 and Table 2; Figure 2). To increase activity and enhance stability, cysteine pairs have been utilized and also replacement of lysine (K) and arginine (R) with the non-natural amino acid, ornithine (O) created innovative structures that are less prone to proteolytic degradation [18,25]. The presence and number of disulfide bonds influenced antifungal activity. The bis-disulfide peptides, RP556 and RP557, exhibited broad-spectrum activity against all fungal isolates evaluated. They were superior to the earlier generation linear dAMP, D4E1, and were far superior to the single disulfide 12 amino acid RP513, and better than the 24 amino acid mono-disulfide dAMP, RP504, especially against *Aspergillus* spp. (Table 1). Iterative substitution of the un-natural amino acid ornithine into the D4E1 template produced peptides with increased proteolytic stability and increased activity. Specific replacement of lysine in RP555 at positions 8 and 10 for ornithine to form RP554, generated enhanced anti-fungal activity, especially against *Aspergillus* spp.

As bacteria often co-infect trauma wounds, the dAMPs were evaluated against both Gram-positive and Gram-negative strains (Table 2). Furthermoreunpub, as was observed with antifungal screening, the structurally confined bis-disulfide peptides, RP556 and RP557 were generally more active than the mono-disulfides RP504 and RP513 and the linear RP554 and RP555 peptides. The exception however was RP553 which generally had similar activity to RP556 and RP557. The distribution of short clusters of hydrophobic and hydrophilic amino acids increased the antimicrobial range of RP553. Unfortunately, the charge and reduced hydrophobicity provided by the ornithine coupled with the linear conformation of RP553, and also RP554, resulted in untoward mammalian cytotoxicity. Our iterative process identified two dAMPs (RP556 and RP557) that have within their 17 amino acid bis-disulfide sequence a unique structure with amphipathic regions that endow them with significant antifungal and antimicrobial activity and limited cytotoxicity to mammalian cells.

These synthetically created peptides have demonstrated increased potency, efficacy, safety, specificity and reduced toxicity in comparison to their natural templates and earlier generation comparators, D4E1 [20]. The dAMPs have direct antibiotic activities in addition to modulating immune responses [16]. dAMPs possess an amphipathic α-helix or β-sheet structure and a net positive charge, critical physicochemical features integral to electrostatically interacting and selectively perturbing the barrier function of the predominantly anionic pathogen membrane. To confirm the mechanism of action via direct membrane disruption, scanning electron microscopy performed on both planktonic and biofilm *C. albicans* was consistent with dAMP-mediated fungal effects via membrane perturbation [17].

Fungi and bacteria do not develop resistance against the dAMPs [16,17,18]. The rapid destruction of pathogenic cells by dAMPs infers a theoretical reduced likelihood of developing bacterial resistance. However, to confirm this, dAMPs were serially passaged against *Candida* and against both *P. aeruginosa* and *S. aureus* at subinhibitory concentrations to ascertain whether resistance, assessed by growth at higher concentrations, occurs [17,18]. Resistance was not observed. Gentamicin and clindamycin developed strong resistance during serial passaging against *P. aeruginosa* and *S. aureus* respectively, whereas the dAMPs did not. Moreover, the generated multidrug resistant strains of *P. aeruginosa* and *S. aureus* were readily susceptible to dAMP treatment.

Early and empiric treatment is required due to the rapid emergence of both bacterial and fungal resistance and resilience due to biofilm formation multi-drug resistant organisms and their associated biofilms play a significant role in the pathogenicity and chronicity of wound infections [26]. The resistance of bacteria and fungi residing in biofilms is due to the biofilm matrix acting as a fortified barrier and the presence of slow-growing microbes with poor metabolic activity, known as persisters. The dAMPs direct perturbation of cellular membranes is effective at eradicating biofilms. RP557 was shown to completely disrupted both preformed and mature MRSA biofilm and *Candida* biofilm [17,18].

Studies demonstrating in vitro antifungal efficacy provided the basis for proceeding with an evaluation of dAMPs in an in vivo fungal infection. The dAMPs RP504, RP554 and RP557 were thus tested for their ability to treat a rodent vulvovaginal candidiasis infection [17]. After three days of daily treatment, each of these dAMPs significantly reduced the CFU/tissue sample by a minimum of 2.5 logs. A dose-response protocol with RP557 demonstrated decreasing CFU/sample with increasing dose levels. A daily dose with a 2% concentration lowered the CFU/sample by more than 4 logs compared to the control samples. Rats with vulvovaginal candidiasis treated with oral fluconazole, a current standard therapeutic agent, had a non-significant reduction in tissue candidiasis infection [17].

The broad spectrum antifungal and antibacterial efficacy of dAMPs, as demonstrated in the above studies via in vitro assays that then translated into significant in vivo efficacy, suggests that these peptides are potential therapeutic agents for the broad-spectrum empiric topical treatment of traumatic wound infections.

## 5. Patents

Jaynes J, Clemens LE, Lopez HW, Martin GR, Woodburn KW, US 10,017,542 B2, issued 10 July 2018, “Antimicrobial Peptides and Methods of Use Thereof.”

Jaynes J, Clemens LE, Lopez HW, Martin GR, Woodburn KW, US 10,548,944, issued 4 February 2020, “Antimicrobial Peptides and Methods of Using the Same.”

## Figures and Tables

**Table 1:** Minimum Inhibitory Concentrations (MICs, μg/mL) against Fungal Strains.

Fungi	D4E1	RP504	RP513	RP553	RP554	RP555	RP556	RP557
*Candida albicans* Y-326	64	8	64	64	32	32	16	16
*Candida albicans* Y-6359	64	4	64	64	32	32	8	4
*Candida parapsilosis* Y-1761	64	8	>128	64	16	32	32	16
*Candida parapsilosis* Y-1763	64	2	>128	32	8	16	2	2
*Candida krusei* Y-27803	64	8	64	32	16	32	16	16
*Candida krusei* Y-27825	64	8	64	32	16	32	32	16
*Aspergillus fumigatus* 9648	128	>128	128	128	64	64	64	64
*Aspergillus fumigatus* 9651	64	>128	>128	64	32	64	64	64
*Aspergillus flavus* MYA-3651	128	>128	128	128	32	128	64	64
*Aspergillus flavus* MYA-1004	128	>128	>128	128	64	>128	64	64
*Absidia corymbifera* NRRL 6251	2	8	32	2	4	4	2	2
*Fusarium solani* NRRL 28548	2	2	16	2	2	2	2	2
*Mucor circinelloides* NRRL 3631	128	>128	32	64	32	64	64	64

MIC, the lowest concentration of test article that shows no growth after a 24 h incubation over the dose range of 2 to 128 µg/mL. Shaded values have been previously reported [17].

**Table 2:** MICs (μg/mL) against Gram-positive & Gram-negative Bacteria.

dAMPs	D4E1	RP504	RP513	RP553	RP554	RP555	RP556	RP557
**Gram-negative bacteria**								
*A. baumannii* 6043	>128	32	64	16	8	16	8	8
*A. baumannii* 6838	8	64	128	8	4	4	8	8
*A. baumannii* ATCC 17978	16	32	>128	4	4	4	8	16
*E. cloacae* 6053	8	32	>128	4	4	2	8	8
*E. cloacae* 6054	4	64	>128	2	2	4	8	8
*K. pneumonia* 6069	16	32	128	4	16	16	8	4
*K. pneumonia* ATCC 10031	32	32	128	4	8	4	8	8
*P. aeruginosa* 6186	16	16	>128	8	8	4	4	4
*P. aeruginosa* ATCC 19660	32	16	>128	8	8	8	8	4
*P. aeruginosa* ATCC 27853	16	16	>128	4	4	4	8	4
**Gram-positive bacteria**								
*S. aureus* B-767	4	32	>128	4	4	8	8	4
*S. aureus* 6061	8	64	>128	8	32	16	16	8
MRSA 6313	8	64	>128	4	32	16	8	4
MRSA 6381	8	64	>128	8	32	16	4	4
MRSA ATCC 33592	8	32	>128	4	32	8	4	4
*S. epidermis* ATCC 51625	4	16	>128	2	2	2	4	8

Shaded values have been previously reported [18].
